# Impact of Simulation-based Learning and Previous Academic Achievement on Clinical Judgment in Nursing Students

**DOI:** 10.31662/jmaj.2025-0299

**Published:** 2025-09-26

**Authors:** Ayako Nishimura, Yuma Ota, Yasuyo Kasahara

**Affiliations:** 1 Faculty of Healthcare, Division of Nursing, Tokyo Healthcare University, Tokyo, Japan

**Keywords:** nursing students, clinical reasoning, clinical judgment, simulation‐based learning, critical thinking

## Abstract

**Introduction:**

Clinical judgment is crucial for nurses because it directly impacts patient safety and outcomes. Simulation is an effective strategy for teaching clinical reasoning and judgment. This study investigated the relationship between simulation performance and foundational knowledge from previous academic subjects, as measured by clinical judgment performance on the Lasater Clinical Judgment Rubric (LCJR). This study aimed to determine the impact of simulation-based learning and previous academic achievement on LCJR scores among nursing students.

**Methods:**

In this cross-sectional quantitative study, a questionnaire was administered to second-year undergraduate nursing students (n = 85) after they completed simulation-based learning. The questionnaire captured self-assessed achievement levels related to the simulation’s learning objectives and previous academic subjects. Statistical analysis included normality tests, Cronbach’s α, Spearman’s correlation, and multiple regression, with simulation and subject achievement as independent variables and LCJR scores as the dependent variable.

**Results:**

Achievement of learning objective 3 (the “expectation to initial grasp”) within the simulation, along with self-reported achievement in physical assessment, critical thinking, and nursing processes from previous academic subjects, significantly affected the LCJR total score and the “Interpreting” dimension. Physical assessment, critical thinking, and nursing processes also significantly impacted the LCJR “Noticing” dimension. Achievement of learning objective 1 (“Gathering information from electronic medical records”) and learning objective 3 affected the LCJR “Responding.”

**Conclusions:**

The achievement of physical assessment, critical thinking, and nursing process in the previous academic subjects affected the total LCJR scores and the scores of Noticing and Interpreting. Emphasizing these areas of nursing education may enhance the students’ clinical judgment skills.

## Introduction

Nurses are required to have clinical reasoning, critical thinking, creative thinking, scientific thinking, and formative critical reasoning skills because of increasingly complex health care environments and expanding professional roles ^(1), (2)^. Clinical reasoning is essential for new nurses and impacts patient outcomes ^(3)^. Good clinical reasoning positively impacts patient outcomes, whereas poor clinical reasoning can lead to failure to recognize deterioration in a patient’s condition, thereby compromising patient safety ^(4)^. Poor clinical reasoning among nurses leads to a major cause of adverse patient status changes, failure to recognize and intervene timely, inadequate management of complications ^(4)^, shock during the transition from nursing students to professional nurses ^(5)^, and nursing diagnostic errors ^(6)^.

In education, strategies aimed at developing appropriate decision-making, clinical reasoning, and clinical judgment based on decision-making ^(7)^, as well as safe and effective clinical judgment that contributes to nursing outcomes and quality of care, are essential ^(8)^. Simulation learning has been proposed as the most effective strategy for teaching clinical reasoning and judgment outside of clinical practice ^(5)^.

In a systematic review evaluating the clinical reasoning of students and health care professionals in practice and simulation ^(9)^, more than half of the studies used the Script Concordance Test or Lasater Clinical Judgment Rubric (LCJR) ^(10), (11)^. As an assessment instrument ^(12)^, the LCJR has been used as an effective tool to directly measure clinical judgment based on a standardized language specific to nursing practice ^(12)^ and has been verified for the assessment of clinical judgment and educational evaluation. In educational evaluations integrating clinical reasoning and judgment into the curriculum ^(13), (14), (15), (16)^, methods for teaching and assessing clinical judgment, the reliability and validity of assessment tools, educational evaluations based on these tools, and the curriculum’s connections with other subjects have been validated.

Basic nursing education in Japan requires the use of clinical judgment models such as the clinical judgment model ^(17)^ and education on clinical judgment. Clinical judgment was specified in the partial revision of the Regulations for Designation of Schools of Public Health Nurses, Midwives, and Nurses in 2022 ^(18)^. However, in basic nursing education, there is no consensus regarding educational methods or the evaluation of clinical judgment and reasoning ^(19), (20)^ nor are there clearly defined learning objectives, evaluation criteria, or achievement levels related to clinical judgment and its connection to specialized foundational subjects.

Therefore, this study examined the impact of achievement levels of nursing students’ simulation-based learnings and previous academic subjects on LCJR.

## Materials and Methods

### Research design

This was a cross-sectional study with quantitative analytical research.

### Research subjects

Of the 110 second-year students enrolled in the Division of Nursing at University A, 95 who participated in simulation-based learning in the “Nursing Process and Clinical Judgment” course received the educational intervention. Eighty-five consenting students were selected as participants.

### Study period

This study was conducted between December 2, 2022 and December 20, 2022.

### Educational intervention (Figure 1)

This study follows the guidelines for reporting evidence-based practice (EBP) educational interventions and teaching as outlined by EBP ^(21)^. Details of the educational interventions applied in this study are described subsequently.

**Figure 1.**
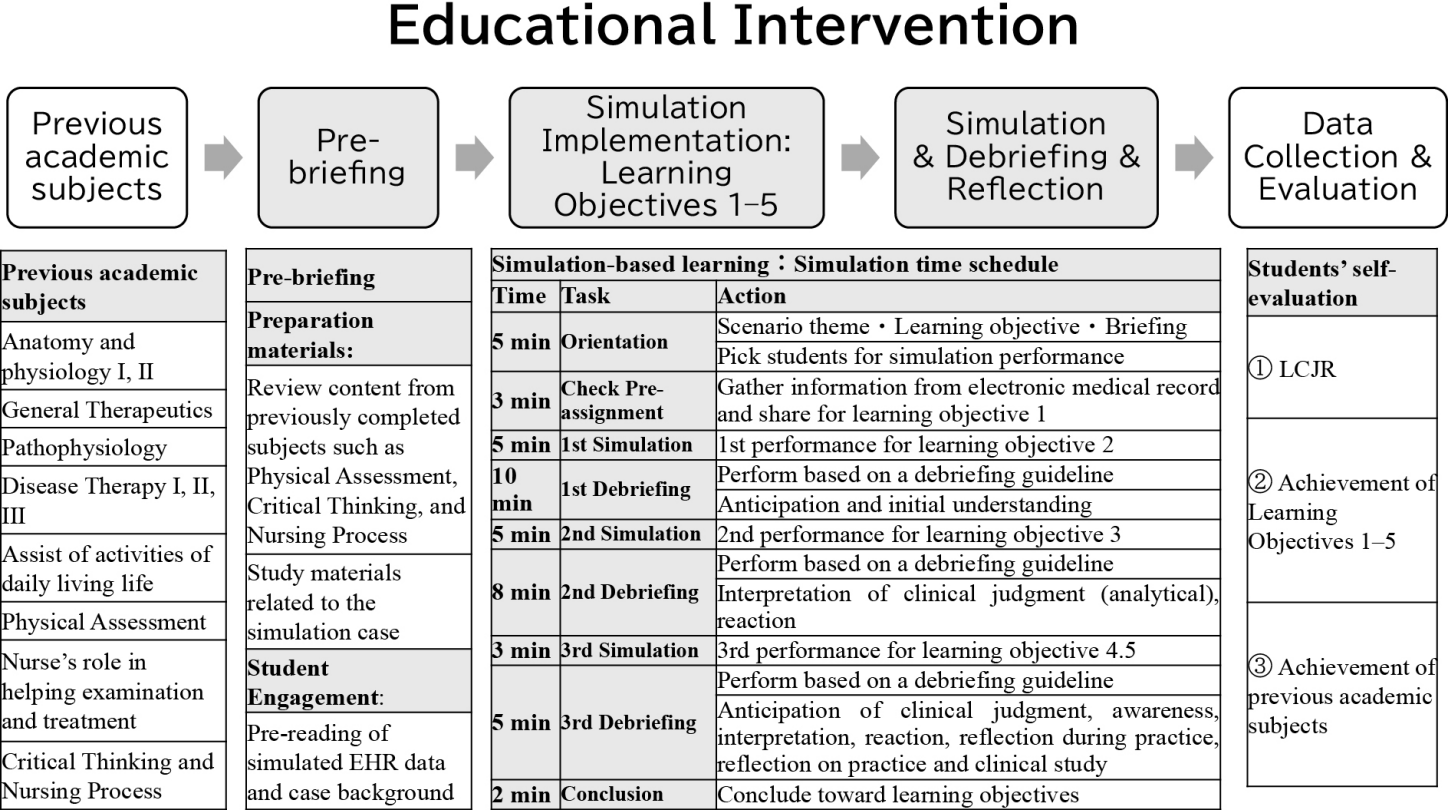
Educational Intervention. This figure shows the overall educational intervention.

#### 1) Applicable simulation-based learning courses as educational interventions

“Nursing Process and Clinical Decision-Making” is a 1-credit (30 hours) course offered in the second semester of the second year and integrates previous academic subjects. The 14th simulation, which is the relevant simulation for this study, is a comprehensive simulation of clinical reasoning and judgment based on the nursing process in which students practice nursing using the knowledge, skills, and attitudes they have acquired.

The students had already completed courses in specialized foundational subjects; this course served as their final lecture and practicum in basic nursing. In addition, they had previously learned the fundamentals of the nursing process, clinical judgment, critical thinking, and clinical reasoning, assessed through examinations evaluating each process.

#### 2) Learning objectives for the simulation-based learning on clinical judgment

Students were given a pre-briefing one week before the start of the class, which included the learning objectives, schedule, pre- and post-assignments, and necessary preparations. The five learning objectives of simulation-based learning on clinical judgment are as follows:

Learning objective 1: [Pre-assignment] Demonstrate the ability to gather relevant information from electronic medical records.

Learning objective 2: Demonstrate an understanding of the anatomy and physiology necessary for clinical judgment.

Learning objective 3: Anticipate the patient’s condition, prioritize observations, and develop an initial understanding.

Learning objective 4: Interpret information obtained through observation and respond appropriately, including using the I-SBAR (Introduction, Situation, Background, Assessment, Recommendation) format report and providing nursing assistance.

Learning objective 5: [Post-assignment] Reflect on outcomes and recognize the clinical judgment process.

#### 3) Educational theories used in educational interventions

The educational interventions used in this study were designed as follows: preparation for the simulation-based learning was based on the checklist of the National Council of State Boards of Nursing (NCSBN) Simulation Guidelines for Prelicensure Nursing Education Programs ^(22)^. The preparation of the simulation-based learning scenarios was based on the Healthcare Simulation Standards of Best Practice Simulation Design ^(23)^. Simulation design features, outcomes, facilitators, and educational practices were organized based on the NLN(National League for Nurses )/Jeffries Simulation Framework ^(24)^. The educational intervention was designed based on Kolb’s Experiential Learning Model ^(25)^ to secure time and opportunities for concrete experiences, reflective observations, and abstract concepts and the Simulation Research Rubric ^(26)^ to ensure simulation studies.

#### 4) Contents of the EBP

Based on the Diabetes Mellitus Standard Medical Treatment Manual 2019 ^(18)^, evidence and case scenarios were incorporated into the decision support tool to include the data necessary for interpreting risks, benefits, and disadvantages ^(27)^. Specifically, this scenario was used to observe the hypoglycemic status and initial responses of a simulated patient hospitalized for diabetes mellitus.

#### 5) Teaching materials, pre-tasks, and learning environment

A learning management system (WebClass; Data Pacific [Japan] Ltd.) was used to administer the pre- and post-assignments for simulation-based learning. The pre-assignment was delivered through an educational electronic medical record designed to promote the students’ awareness of clinical judgment and to guide them in information gathering. In addition, students were instructed to collect and study the information necessary for the case using the Minds Clinical Practice Guidelines ^(28)^. The pre-assignment was expected to take approximately two hours, including the time required to extract information from the electronic medical records.

The simulations were conducted in an on-campus laboratory. A home-visit nursing bag was prepared for providing first-response care to a patient with diabetes. The bag contained a blood pressure cuff, stethoscope, a SpO_2_ (peripheral capillary oxygen saturation) monitor, a penlight, and a stopwatch, as well as personal protective equipment. The simulation involved one group (hereinafter referred to as “G”) of five students, with one faculty member per group serving as facilitator, debriefer, and simulated patient. In addition, one overall facilitator oversaw each set of 30 students (5 students per group × 6 groups = 30 students). The sessions were repeated four times, allowing a maximum of 120 students (30 students × 4 sessions = 120 participants) to participate.

#### 6) Simulation design

The simulation design was developed by a faculty member trained as a designer, facilitator, and debriefer, including completion of the simulation educator step-up course, and who has over nine years of experience in simulation design and instructor training. A pilot test was conducted with five faculty members responsible for the simulation to refine the scenarios.

#### 7) Scenario simulation time schedule and debriefing guide

The scenario was designed to require nursing students to generate multiple hypotheses regarding acute and chronic complications of hypoglycemia and hyperglycemia. It involved diagnostic judgment to assess the urgency and severity of hypoglycemic symptoms by evaluating and accepting or rejecting these hypotheses, as well as therapeutic judgment to determine safe and timely interventions for hypoglycemic symptoms. Simulation-based learning was designed to integrate knowledge and experience from specialized foundational courses and basic nursing in the specialty field, aiming to develop the students’ ability to systematically and holistically understand and critically analyze clinical situations.

Students were expected to report clinical outcomes at the appropriate time using institutional systems and evaluate whether their reports contributed positively to patient outcomes.

The debriefing guide was based on the framework of Tanner’s clinical judgment model of “Noticing,” “Interpreting,” “Responding,” and “Reflecting,” and the content required analytical reasoning patterns based on previous academic subjects for interpretation.

### Data collection

Data were collected using a questionnaire administered after the completion of simulation-based learning. Consenting students responded to the achievement level and LCJR ^(11)^ of the simulation-based learning and previous academic subjects.

#### 1) LCJR (11 items)

The LCJR has been used in many countries to assess the development of clinical judgment in learners ^(12)^, and its Japanese version has been developed and validated for reliability and validity ^(29)^. Permission was obtained from the developer of the Japanese version for its use in this study. The LCJR consists of 11 evaluation items organized into four phases, yielding a total score. Each evaluation item is rated on a four-level scale: exemplary, accomplished, developing, and rudimentary. Scores are assigned as follows: 4 for exemplary, 3 for accomplished, 2 for developing, and 1 for rudimentary.

#### 2) Achievement level of simulation-based learning and previous academic subjects

The achievement levels of simulation-based learning and previous academic subjects were self-assessed using a 10-point scale, ranging from “achieved” to “not achieved.” Based on an international literature review identifying subjects that influence clinical judgment, the previous academic subjects selected included those in the specialized foundational fields defined by Japanese designation rules, as well as basic nursing subjects essential for understanding and responding to simulation cases. Specifically, the specialized foundational subjects were “Anatomy and Physiology I and II,” “General Therapeutics,” “Disease Therapy I, II, and III,” and “Pathophysiology.” The specialty field included eight subjects, such as “Assist of Activities of Daily Living,” “Physical Assessment,” “Nurse’s Role in Helping Examination and Treatment,” and “Critical Thinking and the Nursing Process.”

### Analysis methods

The required sample size for this study was calculated using G*Power ^(30)^ based on the significance level, statistical power, effect size, and number of independent variables. The significance level (α) was set at 0.05, statistical power (1-β) at 0.90, effect size at 0.35, and the number of independent variables at 13. The final sample comprised 77 respondents.

The distribution of responses to the LCJR reflected its characteristics and revealed response biases among nursing students. Each LCJR item was tested for normality using the Kolmogorov-Smirnov test (p = 0.003) and summarized using medians and interquartile ranges (IQRs) due to non-normality. Items with missing responses were excluded from the analysis. Cronbach’s α coefficients were calculated to assess the internal consistency of LCJR items across the four phases of the clinical judgment model.

Spearman’s correlation coefficients were calculated between the achievement level of the simulation-based learning and previous academic subjects, the total LCJR scores between each variable, and the scores of the four phases. In addition, a multiple regression analysis using the forced entry method was conducted with the achievement level of the simulation and previous academic subjects as independent variables and LCJR scores as the dependent variable. Multicollinearity among the independent variables was checked using the variance inflation factor, with a criterion of <10.

EZR(Easy R) ^(31)^ was used for all statistical analyses, with a significance level of 5%.

### Ethical consideration

This study was approved by the ethics committee. To protect the rights of participants, we ensured that participation was voluntary, participants could withdraw their consent at any time during the study, and no academic disadvantages would result from their decision. To safeguard participant privacy, we explained that survey responses were anonymous, researchers would not know the identity of respondents, and participation would have no impact on academic records. Consent was obtained through an electronic, unsigned, self-administered questionnaire; only responses from participants who provided consent were included in the study data.

## Results

Eighty-five responses were obtained for all questions (valid response rate: 89.5%).

### Frequency distribution of LCJR

The frequency distribution and descriptive statistics of the LCJR are presented in Table 1. All 11 assessment items consisting of the four phases of the LCJR were median 2 (IQR 2-3) (Table 1).

**Table 1.** LCJR Frequency Distribution Table.

						n=85
	Exemplary n (%)	Accomplished n (%)	Developing n (%)	Beginning n (%)	Mean (SD)	Median [IQR]
Effective noticing involves:						
Focused observation	2(2.35)	39 (45.88)	44 (51.76)	0 (0)	2.51 (0.55)	2 [2-3]
Recognizing deviations from expected patterns	2 (2.35)	37 (43.53)	44 (51.76)	2 (2.35)	2.46 (0.59)	2 [2-3]
Information seeking	2 (2.35)	34 (40.00)	49 (57.65)	0 (0)	2.45 (0.55)	2 [2-3]
Effective interpreting involves:						
Prioritizing data	3 (3.53)	36 (42.35)	46 (54.12)	0 (0)	2.49 (0.57)	2 [2-3]
Making sense of data	2 (2.35)	27 (31.76)	56 (65.88)	0 (0)	2.36 (0.53)	2 [2-3]
Effective responding involves:						
Calm, confident manner	1(1.18)	27 (31.76)	53 (62.35)	4 (4.71)	2.29 (0.57)	2 [2-3]
Clear communication	4 (4.71)	36 (42.35)	42 (49.41)	3 (3.53)	2.48 (0.65)	2 [2-3]
Well-planned intervention/flexibility	1 (1.18)	27 (31.76)	55 (64.71)	2 (2.35)	2.32 (0.54)	2 [2-3]
Being skillful	0 (0)	22 (25.88)	60 (70.59)	3 (3.53)	2.22 (0.50)	2 [2-3]
Effective reflecting involves:						
Evaluation/Self-analysis	2 (2.35)	31 (36.47)	51 (60.00)	1 (1.18)	2.40 (0.56)	2 [2-3]
Commitment to improvement	1 (1.18)	28 (32.94)	56 (65.88)	0 (0)	2.35 (0.50)	2 [2-3]

*The Japanese version of the LCJR was used with permission from Dr. Hosoda. *IQR: interquartile range; LCJR:Lasater Clinical Judgment Rubric; SD: standard deviation.

### Achievement level of simulation-based learning and previous academic subjects

Table 2 shows the descriptive statistics of the achievement levels of simulation-based learning and previous academic subjects. All seven subjects, except for “Assist of activities of daily living life,” were median 6 (IQR 5-7). The same results were obtained for learning objectives 1-5 in simulation-based learning.

**Table 2.** Achievement Level of Simulation-based Learning and Previous Academic Subjects.

		n = 85
Achievment level in simulation-based learning (n = 85)		Median [IQR]
Learning Objective 1: [Pre-assignment] Demonstrate the ability to gather relevant information from electronic medical records.		6 [5-7]
Learning Objective 2: Demonstrate an understanding of the anatomy and physiology necessary for clinical judgment.		6 [5-7]
Learning Objective 3: Anticipate the patient’s condition, prioritize observations, and develop an initial understanding.		6 [5-7]
Learning Objective 4: Interpret information obtained through observation and respond appropriately, including using the I-SBAR report and providing nursing assistance.		6 [5-7]
Learning Objective 5: [Post-assignment] Reflect on outcomes and recognize the clinical judgment process.		6 [5-7]
**Achievement level of previous academic subjects**		**Median [IQR]**
Specialized foundational subjects	Anatomy and physiology I, II	6 [5-7]
	General Therapeutics	6 [5-7]
	Pathophysiology	6 [5-7]
	Disease Therapy I, II, III	6 [5-7]
Specialty field	Assist of activities of daily living life	7 [6-8]
	Physical Assessment	6 [5-7]
	Nurse’s role in helping examination and treatment	6 [5-7]
	Critical Thinking and the Nursing Process	6 [5-7]

*10-point scale (1: not achieved, 10: achieved) *IQR: interquartile range.

### Reliability of LCJR

Cronbach’s α were 0.905, 0.862, 0.908, 0.902, and 0.822 for the LCJR total score and the four phases: Noticing, Interpreting, Responding, and Reflecting, respectively.

### Relationship between the achievement levels of simulation-based learning and previous academic subjects for LCJR (total score and four phases)

The total LCJR score and scores for its four phases were significantly positively correlated with achievement levels in simulation-based learning and previous academic subjects. Among these variables, all except for the achievement levels in learning objective 1 of the clinical judgment simulation and the Critical Thinking and Nursing Process subject, showed significant positive correlations with the scores of each LCJR phase (Table 3).

**Table 3.** Relationship between the Achievement Levels of Simulation-based Learning and Previous Academic Subjects for LCJR (Total Score and Four Phases).

					n = 85	
	Item	Total LCJR	Noticing	Interpreting	Responding	Reflecting
Achievement levels in simulation-based learning	Learning Objective 1: [Pre-assignment] Demonstrate the ability to gather relevant information from electronic medical records.	0.255 *	0.335 **	0.237 *	0.192	0.220 *
	Learning Objective 2: Demonstrate an understanding of the anatomy and physiology necessary for clinical judgment.	0.311 **	0.302 **	0.284 **	0.289 **	0.322 **
	Learning Objective 3: Anticipate the patient’s condition, prioritize observations, and develop an initial understanding.	0.433 ***	0.439 ***	0.382 **	0.402 ***	0.381 ***
	Learning Objective 4: Interpret information obtained through observation and respond appropriately, including using the I-SBAR report and providing nursing assistance.	0.397 ***	0.402 ***	0.331 **	0.392 ***	0.351 **
	Learning Objective 5: [Post-assignment] Reflect on outcomes and recognize the clinical judgment process.	0.396 ***	0.429 ***	0.330 **	0.366 ***	0.342 **
	**Item**	**Total LCJR**	**Awareness**	**Interpretation**	**Reaction**	**Reflection**
Achievement levels in previous academic subjects	Anatomy and physiology I, II	0.291 **	0.314 **	0.249 *	0.279 **	0.310 **
	General Therapeutics	0.317 **	0.297 **	0.217 *	0.370 ***	0.348 **
	Pathophysiology	0.263 *	0.262 *	0.227 *	0.295 **	0.273 *
	Disease Therapy I, II, III	0.353 ***	0.33 **	0.241 *	0.394 ***	0.384 ***
	Assist of activities of daily living life	0.377 **	0.373 ***	0.345 *	0.379 ***	0.385 **
	Physical Assessment	0.419 ***	0.415 ***	0.386 **	0.428 ***	0.413 ***
	Nurse’s role in helping examination and treatment	0.369 **	0.389 ***	0.314 *	0.399 ***	0.374 ***
	Critical Thinking and the Nursing Process	0.247 *	0.246 *	0.182	0.250 *	0.270 *

*10-point scale (1: not achieved, 10: achieved). *p < 0.05, **p < 0.01, ***p < 0.001. *LCJR:Lasater Clinical Judgment Rubric.

### Multiple regression analysis with LCJR total score as the dependent variable

A multiple regression analysis was conducted using the LCJR total score as the dependent variable, with the achievement levels of simulation-based learning and previous academic subjects, previously identified as significantly correlated, entered as independent variables.

In step 1, a multiple regression analysis was conducted using the forced entry method, with the achievement level of learning objectives 1-5 in simulation-based learning as the independent variable, excluding previous academic subjects. In step 2, we added the level of achievement of previous academic subjects to the independent variables entered in step 1 and checked the change in the goodness of fit of the multiple regression equations in steps 1 and 2. Consequently, the achievement levels of previous academic subjects were added as independent variables.

The goodness of fit for step 2 indicated an improved regression model with an adjusted R^2^ of 0.300. In this model, higher self-assessment scores for “Learning Objective 3: Able to anticipate the patient’s condition, prioritize observations, and develop an initial understanding,” along with higher achievement levels in previous academic subjects, were associated with higher total LCJR scores. No multicollinearity was detected among the variables (Table 4).

**Table 4.** Multiple Regression Analysis with LCJR Total Score as the Dependent Variable.

n = 85					
Lasater Clinical Judgment Rubric (LCJR) Total Score					
		Step 1		Step 2	
		β	VIF	β	VIF
	Achievement levels in simulation-based learning				
	Learning Objective 1	-1.64	5.335	-1.223	7.776
	Learning Objective 2	0.09	6.36	0.378	9.126
	Learning Objective 3	2.239 *	9.601	3.306 *	9.157
	Learning Objective 4	0.08	8.073	-0.775	9.924
	Learning Objective 5	0.829	9.609	0.353	9.101
	R2	0.28***		0.40***	
	Adjusted R2	0.23***		0.30***	
	Achievement levels in previous academic subjects				
Specialized basic field	Anatomy and physiology I, II			0.314	6.419
	General Therapeutics			0.385	9.091
	Pathophysiology			0.026	9.117
	Disease Therapy I, II, III			1.162	7.441
Specialty field	Assist of activities of daily living life			0.021	8.831
	Physical Assessment			2.483 *	9.713
	Nurse’s role in helping examination and treatment			0.354	8.000
	Critical Thinking and Nursing Process			1.740 *	4.768

*p < 0.05, **p < 0.01, ***p < 0.001. β: Standard Partial Regression Coefficient, R2: Determination Coefficient, VIF: Variance Inflation Factor. All 10-point scale (1: not achieved, 10: achieved)

### Multiple regression analysis with LCJR’s noticing, interpreting, responding, and reflecting as dependent variables

The results indicated that higher achievement levels in Physical Assessment and Critical Thinking and Nursing Process were associated with higher scores in the Noticing phase (Table 5). The results for learning objective 3 in the simulation-based learning indicated that higher levels of achievement in Physical Assessment and Critical Thinking and Nursing Process were associated with higher Interpreting scores. Furthermore, higher levels of achievement in learning objective 1, “Collecting information from electronic medical records,” and learning objective 3 in the simulation-based learning were associated with higher Responding scores. In addition, no independent variable significantly increased Reflecting.

**Table 5.** Multiple Regression Analysis with LCJR’s “Noticing,” “Interpreting,” “Responding,” and “Reflecting,” as Dependent Variables.

										n = 85
	Lasater Clinical Judgment Rubric (LCJR) Total Score・4 Phases									
	Total Score		Noticing		Interpreting		Responding		Reflecting	
Achievement levels in simulation-based learning	β	t	β	t	β	t	β	t	β	t
Learning Objective 1	-1.223	-1.395	-0.303	0.653	-1.223	-1.689	0.784 *	2.064	-0.303	-1.654
Learning Objective 2	0.378	0.377	0.226	-1.155	0.378	1.425	0.205	0.518	0.226	1.076
Learning Objective 3	3.306 *	2.128	0.363	1.426	3.306 *	2.490	0.909 *	2.124	0.363	1.603
Learning Objective 4	-0.775	-0.725	-0.171	-1.452	-0.775	-0.818	0.051	0.121	-0.171	-0.767
Learning Objective 5	0.353	0.332	-0.033	-1.394	0.353	0.026	-0.058	-0.138	-0.033	-0.149
Achievement levels in prior academic subjects										
Anatomy and physiology I, II	0.314	0.383	0.147	0.763	0.314	0.701	-0.144	-0.444	0.147	0.859
General Therapeutics	0.026	0.027	0.052	0.905	0.026	0.541	0.338	0.912	0.052	0.267
Pathophysiology	1.162	1.323	0.257	1.485	1.162	0.979	0.328	0.946	0.257	1.403
Disease Therapy I. II. III	0.385	0.412	0.060	1.501	0.385	0.181	-0.065	-0.177	0.06	0.307
Assist of activities of daily living life	0.021	0.022	0.065	0.406	0.021	0.779	-0.083	-0.213	0.065	0.315
Physical Assessment	2.483 *	2.133	0.382 *	2.164	2.483 *	2.009	0.846	1.841	0.382	1.572
Nurse’s role in helping examination and treatment	0.354	0.380	0.038	0.258	0.354	0.066	0.255	0.695	0.038	0.196
Critical Thinking and Nursing Process	1.740 *	2.230	0.223 *	2.123	1.740 *	3.003	0.523	1.696	0.223	1.370
R2	0.41***		0.34***		0.40***		0.41***		0.34**	
Adjusted R2	0.30 ***		0.22 ***		0.30***		0.30***		0.22**	

*p < 0.05, **p < 0.01, ***p < 0.001. β: Standard Partial Regression Coefficient, R2: Determination Coefficient, t: t-value. All 10-point scale (1: not achieved～10: achieved).

## Discussion

### Correlation between achievement levels in the simulation-based learning and previous academic subjects for the four phases of LCJR

This study found that all four phases of the LCJR were correlated with achievement levels in simulation-based learning and previous academic subjects. These findings suggest that clinical judgment, as measured by the LCJR, is related to achievement in specialized foundational subjects, basic nursing, and simulation-based learning. In addition, although no correlation was observed between the previous academic subject Critical Thinking and Nursing Process and the Interpreting phase, which corresponds to the analytical reasoning aspect of clinical judgment, significant correlations were found between achievement levels in learning objectives 2, 3, 4, and 5 of simulation-based learning and the Interpreting phase. This suggests that simulation-based learning likely enhances analytical reasoning in clinical judgment.

The LCJR was selected for this study because it is one of the few tools specifically developed to assess clinical judgment in nursing and has established reliability and validity. Unlike knowledge-based assessments such as the Script Concordance Test, the LCJR evaluates learners’ performance in real-world or simulated clinical scenarios, aligning with experiential learning theory. However, this method is limited by its subjective nature and reliance on observer scoring. Future studies could compare it with objective structured clinical examination tools or multi-source feedback frameworks.

Previous research has shown that thought processes leading to clinical reasoning occur throughout all phases of the nursing process ^(32)^ and that analytic reasoning patterns are influenced by repeated learning of assessment and the nursing process as a foundational skill for assessment, critical thinking, and clinical reasoning and judgment ^(15), (16)^. We hypothesized that the repetitive learning of previous academic subjects by students also influenced their analytical reasoning patterns in clinical judgment.

### Impact of the achievement levels of clinical judgment simulation and previous academic subjects on LCJR total scores

In this study, higher achievement levels in learning objective 3, “Anticipation to Initial Understanding,” and in the previous academic subjects Physical Assessment and Critical Thinking and the Nursing Process were associated with higher total LCJR scores. The judgment domain of clinical judgment includes diagnostic, therapeutic, and ethical judgments ^(33)^. The nursing process involves multiple aspects of clinical reasoning and judgment ^(33)^. Thinking processes encompass the nursing process; clinical reasoning; various types of thinking, such as exhaustive methods, deductive reasoning, hypothetical deduction, reasoning using Bayes’ theorem, algorithms, pattern recognition, and the dual-process diagnostic reasoning model ^(34), (35)^; critical thinking (including systematic data collection, data examination, and hypothesis testing); and other essential assessment elements that impact clinical judgment and decision-making domains ^(36)^. These results suggest a relationship between the LCJR and Critical Thinking and Nursing Process. It is likely that the LCJR, Physical Assessment and Nursing Process, and learning objective 3 were influenced because learning objective 3, and the previous academic subject, Physical Assessment and Nursing Process, require students to form hypotheses based on necessary information collected.

Tanner states that “intuitive reasoning in clinical judgment models requires experience as a nurse” ^(17)^, and Gordon et al. ^(33)^ reported that “beginner students have difficulty with pattern recognition.” Based on the results of this study, the total LCJR score, as well as the Noticing and Interpreting phases, were influenced by Physical Assessment and Critical Thinking and Nursing Process, whereas the Responding phase was influenced by learning objective 1, “Gather appropriate information from the electronic medical records.” These findings suggest the need for educational strategies that foster Noticing through previous information gathering from electronic health records, patient observation via physical assessment, assessment through the nursing process, and critical appraisal through critical thinking. In other words, for beginner students in basic nursing education, appropriate Noticing requires not only intuitive reasoning, such as pattern recognition, but also analytical reasoning supported by comprehensive and systematic examination methods. Clinical judgment must be developed through repeated learning in lectures, exercises, and practical practicum.

This study suggests that the nursing process, rooted in systematic and comprehensive information collection from the initial Noticing phase, with physical assessment serving as its foundation, and supported by analytical reasoning methods such as the thorough method of exhaustion and critical thinking, influences the total LCJR score and the Noticing and Interpreting phases. Based on these findings, we conclude that clinical judgment depends on the nursing process, judgment domains, clinical reasoning and thinking patterns, and critical thinking. Furthermore, students’ previous academic achievement, particularly, in subjects such as Physical Assessment and Critical Thinking, strongly influences simulation outcomes. Future simulation designs may benefit from a scaffolded approach that integrates review of previous knowledge, ensuring students enter simulations with a shared baseline and can effectively apply their foundational learning. Integrating clinical judgment into basic nursing education requires curriculum design that considers the relationship and relative importance of Critical Thinking, Physical Assessment, and Nursing Process, including previous information gathering.

Critical thinking, clinical reasoning, and especially analytic reasoning are emphasized equally across all health care professional education programs. These cognitive abilities are essential for ensuring patient safety and delivering high-quality care, regardless of profession. Therefore, the educational strategies described in this study, particularly, those fostering analytical thinking through simulation, may be applicable to medical and other health professional training. However, because the LCJR was originally developed to assess clinical judgment specific to nursing practice, caution is warranted when applying it directly to other health care disciplines. Further research is necessary to identify appropriate assessment tools and evaluation frameworks tailored to the unique judgment processes of each field.

### Limitations and future issues of this study

One limitation of this study is that it was conducted at a single institution, which may limit the generalizability of the findings. Therefore, multi-site or longitudinal studies are needed to validate the relationship between simulation-based learning and clinical judgment development across diverse educational contexts.

Because achievement levels and LCJR scores were based on the students’ self-evaluations, the possibility of the Dunning-Kruger effect cannot be excluded ^(37)^. Further studies, including peer evaluations, are needed.

### Conclusions

This study aimed to determine the impact of nursing students’ achievement levels in simulation-based learning and previous academic subjects on their LCJR scores.

The results showed that achievement in the previous academic subjects Physical Assessment, Critical Thinking, and Nursing Process influenced the total LCJR score and the scores for the Noticing and Interpreting phases. These findings suggest that Physical Assessment, Critical Thinking, and Nursing Process key subjects within specialized foundational and specialty fields significantly influence clinical judgment among nursing students in basic nursing education. Moreover, Anticipation to Initial Understanding appears to be a foundational component of the clinical judgment process. To strengthen basic clinical judgment skills, it is recommended that clinical reasoning and judgment be integrated into the curriculum, emphasizing systematic and comprehensive information gathering during the initial noticing phase, physical assessment as the basis for noticing, and nursing processes grounded in critical thinking, which, together, support analytical reasoning and critical appraisal.

## Article Information

### Acknowledgments

Informed consent was obtained from all participants involved in this study.

### Author Contributions

Ayako Nishimura took a role of project administration acquiring funding and resources; conceptualized, designed and conducted the study; designed the educational content; analyzed and interpreted the data including data curation; and prepared the manuscript.

Yuma Ota designed the educational content, designed and conducted the study, analyzed and interpreted the data including data curation, and critically reviewed and edited the manuscript.

Yasuyo Kasahara designed the educational content, designed and conducted the study, and critically reviewed and edited the manuscript.

### Conflicts of Interest

None

### Institutional Review Board Approval Code and Name of the Institution

This study was approved by the Ethics Review Committee of Tokyo Healthcare University (approval code: 32-43C).

### Appendix

Part of this paper was presented at the 55th Annual Meeting of the Japanese Society for Medical Education.
